# Synthesis of a Pyrrolo[1,2-*a*]quinazoline-1,5-dione Derivative by Mechanochemical Double Cyclocondensation Cascade

**DOI:** 10.3390/molecules27175671

**Published:** 2022-09-02

**Authors:** Vanessza Judit Kolcsár, György Szőllősi

**Affiliations:** 1 Department of Organic Chemistry, Faculty of Science and Informatics, Institute of Chemistry, University of Szeged, Dóm tér 8, 6720 Szeged, Hungary; 2 ELKH-SZTE, Stereochemistry Research Group, University of Szeged, Eötvös utca 6, 6720 Szeged, Hungary

**Keywords:** anthranilamide, ethyl levulinate, Amberlyst^®^ 15, Brønsted acid, cascade reaction, mechanochemistry, quinazolinone, ball mill

## Abstract

*N*-heterocyclic compounds, such as quinazolinone derivatives, have significant biological activities. Nowadays, as the demand for environmentally benign, sustainable processes increases, the application of compounds from renewable sources, easily separable heterogeneous catalysts and efficient, alternative activation methods is of great importance. In this study, we have developed a convenient, green procedure for the preparation of 3a-methyl-2,3,3a,4-tetrahydropyrrolo[1,2-*a*]quinazoline-1,5-dione through a double cyclocondensation cascade using anthranilamide and ethyl levulinate. Screening of various heterogeneous Brønsted acid catalysts showed that Amberlyst^®^ 15 is a convenient choice. By applying mechanochemical activation in the preparation of this *N*-heterotricyclic compound for the first time, it was possible to shorten the necessary time to three hours compared to the 24 h needed under conventional conditions to obtain a high yield of the target product.

## 1. Introduction

The pharmaceutical importance of *N*-heterocyclic compounds is indisputable [1]. Due to the biological activities of quinazolinone derivatives, such as anticancer, diuretic and antibacterial properties, these molecules caught the attention of organic chemists [2,3,4,5,6,7,8]. Among these, 2,3-dihydroquinazolin-4(1*H*)-ones are privileged building blocks in drug design, which are facilely prepared by the cyclocondensation of anthranilamides and aldehydes or ketones [2,3,4,9]. Various catalysts were used in these cyclocondensations, such as strong bases [10,11], strong mineral or weak organic Brønsted acids [10,12,13,14,15,16], sulfonic acids [4,10,17,18,19,20,21], Lewis acids [22,23,24,25,26,27,28,29,30] and ammonium salts [31,32].

Recent trends in the fine chemical industry require the development of sustainable, environmentally benign processes; thus, in the preparation of 2,3-dihydroquinazolin-4(1*H*)-ones, the application of catalyst- or solvent-free methods [33,34,35,36] and/or employing easily separable heterogeneous catalysts also became widely investigated [37,38,39,40,41,42,43,44,45,46,47]. Among the latter materials, surface-bonded sulfonic, sulfamic or sulfuric acids are privileged heterogeneous catalysts [48,49,50,51,52,53,54,55,56]. Methods replacing anthranilamide with compounds in situ transformed to 2-aminobenzamides, such as isatoic anhydride, 2-aminobenzonitrile, 2-nitro or 2-azidobenzamides, were also applied in cascade processes leading to 2,3-dihydroquinazolin-4(1*H*)-ones [13,57,58,59,60,61,62,63]. Besides their pharmaceutical use, 2,3-dihydroquinazolin-4(1*H*)-ones are easily transformed to quinazolinones by subsequent one-pot oxidation [11,19,20,64,65]. Moreover, the use of additionally functionalized carbonyl compounds allows the preparation of tricyclic hydroquinazoline derivatives through another consecutive one-pot cyclocondensation step [66,67,68]. Among the carbonyl compounds used in such cascade reactions, γ-keto carboxylic acids and esters were applied to obtain pyrrolo[1,2-*a*]quinazoline-1,5-dione derivatives [68,69,70]. As levulinic acid is a platform molecule obtained from biomass and its esterification can be activated by microwave in a solventless organocatalytic system [71,72,73], these allow the sustainable preparation of such *N*-heterotricyclic products using renewable resources.

On the other hand, advancements in the development of environmentally friendly methods were achieved by the application of alternative energy transmissions [74,75]. Among these, mechanochemical activation became widespread due to its operational simplicity and broad applicability [76,77,78,79,80]. Mechanochemical reactions performed in easily available mixer mills may be carried out in a solventless manner or using a minor amount of liquid for ensuring the proper energy transmission and mixing, termed liquid-assisted grinding (LAG). A wide range of milling conditions can be optimized to achieve high efficiency in various organic reactions, such as the agitation speed, the milling time and the size, number and material of the grinding media [81,82]. Finding appropriate conditions to efficiently carry out certain organic transformations by mechanochemical activation is still a challenging task and requires detailed studies. A variety of organic reactions were carried out by mechanochemical activation at a laboratory scale [76,77,78,79,80,81,82,83,84,85]. These studies indicated that, in most of these reactions, the time can be decreased significantly compared to conventional batch systems. Studies on mechanochemical reactions were also extended to cascade processes, resulting in the formation of valuable heterocyclic compounds [86,87,88,89]. Initial attempts to use mechanochemical activation in catalytic cyclocondensations leading to 2,3-dihydroquinazolin-4(1*H*)-ones were carried out in mortars by grinding the reaction components often followed by heating the mixtures [90,91,92,93,94]. Later, with the widespread application of ball mills in organic synthetic procedures, reactions of anthranilamide and aldehydes or ketones were also efficiently carried out in mixer mills, either solventless or in aqueous media and catalyst-free or catalyzed by potassium iodide or iodine [95,96]. However, the applicability of the mechanochemical activation in cascade double cyclocondensations of anthranilamide and bifunctional carbonyl compounds, to our knowledge, has not yet been explored.

Having in sight the increased importance of one-pot reactions [97], our present study aimed at developing an economic, green and sustainable process for the preparation of a tricyclic pyrrolo[1,2-*a*]quinazoline-1,5-dione derivative by two consecutive cyclocondensations occurring upon reacting anthranilamide and ethyl levulinate. For this, we attempted to use solid acids as heterogeneous catalysts and mechanochemical activation.

## 2. Results and Discussions

### 2.1. Catalytic Cascade Reaction of Anthranilamide and Ethyl Levulinate in the Batch System

**Scheme 1:**
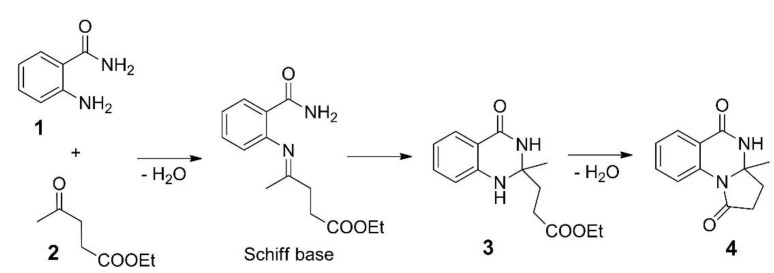
The reaction of anthranilamide (**1**) and ethyl levulinate (**2**) resulting in the formation of 2,3-dihydroquinazolin-4(1*H*)-one (**3**) and pyrrolo[1,2-*a*]quinazoline-1,5-dione (**4**) derivatives.

The condensations of anthranilamide (**1**) and aldehydes or ketones occur through the formation of the corresponding Schiff base followed by a ring-closing step with the participation of the amide moiety (Scheme 1). Products with three condensed rings may be achieved under appropriate conditions with bifunctional carbonyl compounds, such as ethyl levulinate (**2**), as shown in Scheme 1. Thus, in the reaction of **1** and **2** the quinazolinone derivative **3** is formed which, via a second ring-closing step, provides the three-fused ring-containing product **4**. Our initial goal was to choose an appropriate commercial heterogeneous catalyst which may ensure the formation of product **4** under convenient conditions. The study was carried out in a solventless manner in a batch system through magnetic stirring. To ensure proper mixing of the components, 1.5 equivalent (eq) **2** was applied. Measurements carried out without a catalyst showed that, at low temperature (60 °C), small conversion (**Conv**) of **1** could be achieved and that the second ring-closing step took place in a small ratio (see the selectivity of **4** (**S4**), Table 1, entry 1). However, the presence of the Schiff base was not detected at a significant amount, thus its formation rate may be the limiting step of **3** production. Increasing the reaction temperature (90 °C) had a positive effect on the conversion; however, production of **4** still resulted in a low yield (entry 2).

Next, we employed various commercial Brønsted acidic catalysts, which all provided high conversions after 24 or 48 h, some even at 60 °C. The selectivities highly depended on the properties of the applied catalyst. Silica gel and acid-treated montmorillonite clays (Mont K10, Mont KSF) (entries 3–6) were not able to provide high **4** selectivities. On the other hand, catalysts bearing sulfonic acid groups were active in catalyzing the formation of the aimed pyrrolo[1,2-*a*]quinazolinedione derivative (entries 7–14). The application of *p*-toluenesulfonic acid (*p*-TsOH) provided high **S4** only under harsher reaction conditions (90 °C). The propylsulfonic polysiloxane resin (Deloxan^®^ ASP) and the perfluorinated resin bearing sulfonic acid groups (Nafion™ NR50) were also efficient but, similarly to the *p*-TsOH, a higher temperature was necessary to obtain **4** (entries 9–12). The best results were provided by the polystyrene-based sulfonic acid-functionalized Amberlyst^®^-type catalysts (entries 13, 14), both of which afforded full conversions and close to exclusive formation of **4** at 60 °C. Based on these results, we choose to use Amberlyst^®^ 15 in our further investigations.

Examination of the temperature effect was carried out both without a catalyst and with Amberlyst^®^ 15 (see Supplementary Materials, Figure S1). In the absence of a catalyst, a temperature of 90 °C was necessary to achieve close to full conversion; however, **S4** was low over the investigated temperature range, i.e., thermally the second ring-closing step occurred with a low rate. In contrast, by applying Amberlyst^®^ 15, **1** was completely transformed and **S4** increased by raising the temperature, approaching 100% at 60 °C. Thus, under the conditions used in this study, the second cyclization step required the use of an acid catalyst. The same conclusion may be drawn from results regarding the influence of the catalyst amount (Supplementary Materials, Figure S2). At 60 °C, at least 100 mg Amberlyst^®^ 15 was necessary to obtain the desired product **4** in high proportion. Amberlyst^®^ 15 is available from a commercial source in bead-like form. We have powdered it by pre-grinding the material in a ball mill for 10 min to increase its exposed surface sites (denoted as Amberlyst^®^ 15P). By this method, the activity of the catalyst in the second cyclization could be increased significantly, reaching almost 30% higher selectivity of **4** compared to the reaction with the same amount (50 mg) of the commercial form (Figure S2).

Based on the results achieved with Amberlyst^®^ 15P, we further optimized the reaction conditions using this material (Table 2). Decreasing the excess of the reactant to 1.1 eq resulted in lower **4** selectivity, suspected to be due to less efficient mixing of the slurry (Table 2, entries 1, 2). To improve the mixing of the components, the missing volume of **2** was replaced with methanol (MeOH). Close to full conversion and high **S4** were obtained (entry 3). To further study the role of the catalyst, purified **3** (resulting from previous experiments) was used as the starting compound under identical conditions, both without (entry 4) and with Amberlyst^®^ 15P (entry 5). In this case, the second ring-closing step did not occur unless the solid acid was present.

**Scheme 2:**
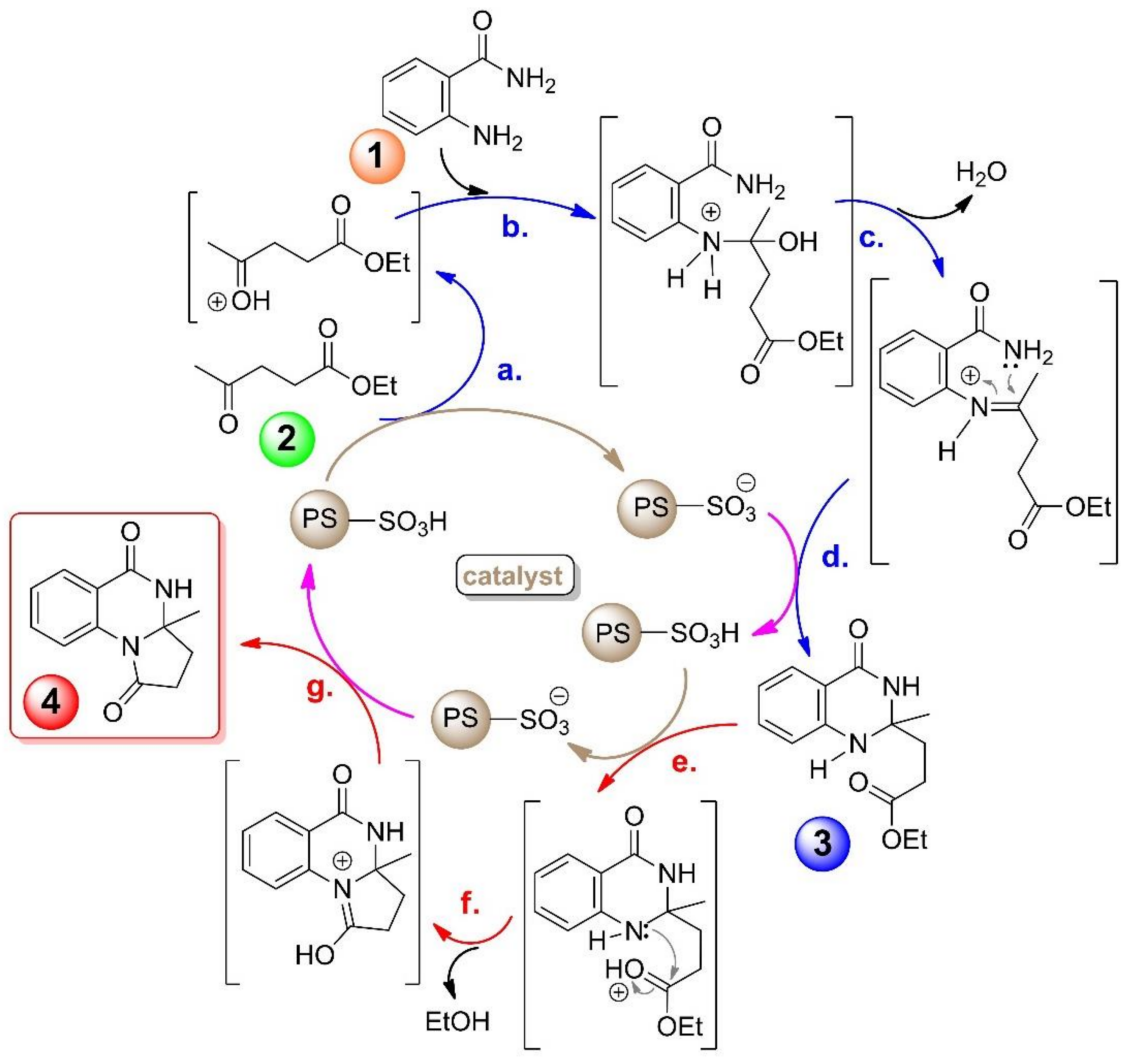
The reaction mechanism of the cascade reaction: blue arrows are the catalytic steps which occurred thermally (**a**.–**d**.) and the red arrows are steps requiring an acid catalyst (**e**.–**g**.).

According to the above, the two cyclocondensation steps of this cascade reaction can be carried out neat, using as little as 1.1 eq **2** by applying a small amount of MeOH and pre-milled Amberlyst^®^ 15 catalyst. The use of MeOH does not diminish the environmentally benign aspect of the method as it is among the organic solvents recommended for use even by some pharmaceutical companies [98]. Based on the obtained results so far and the known mechanism of the cyclocondensations [9,13,23,37,68,70], we could draw conclusions about the activation of the steps, as presented in Scheme 2. We observed that the first cyclization of the cascade reaction leading to **3** can be thermally promoted. Without using a catalyst at 60 °C, 40% conversion was obtained, which increased to 95% at a higher (90 °C) temperature. The product mixture mostly contained the intermediate product **3**, but the formation of a small amount of **4** was also observed, which shows that heating may also promote the second step (Table 1, entries 1, 2). The effect of heating seemed negligible starting from **3** (Table 2, entry 4); however, by introducing Amberlyst^®^ 15 into the system, intramolecular amide formation was accelerated (Table 2, entry 5). According to these findings, the first part of the reaction marked with blue arrows on Scheme 2 (steps **a**.–**d**.) can be promoted by heat as well as acid catalysts. Intramolecular amide formation, on the other hand, is mainly promoted by the acid catalyst (red arrows, steps **e**.–**g**.); thus, in our further studies, the use of a catalyst was necessary to obtain the target compound **4**.

### 2.2. Mechanochemical Catalytic Cascade Reaction of Anthranilamide and Ethyl Levulinate

To make further steps towards the development of a sustainable method, we aimed to carry out the neat reaction of **1** and **2** in a ball mill. Based on the mechanochemical organic reactions recently reported in the literature [99,100], we started our experiments by applying grinding balls of different sizes (Table 3). The number of grinding beads was determined to have similar total volumes in each measurement.

Balls with a bigger diameter (Ø 15 and 12 mm) may provide high collision energy; however, only one piece fits into the jar without hindering another’s movement. Thus, only a low collision number can be achieved. Better **4** selectivity was observed using the Ø 15 mm ball (Table 3, entry 1) than applying the Ø 12 mm ball (entry 2), which shows the importance of collision energy in the second cyclization step. Using 25 pieces (pcs) of Ø 5 mm grinding balls provided an even better result (entry 3). In this case, the higher collision number compensated for the decreased collision energy. However, the use of 125 pcs of Ø 3 mm beads was not efficient, probably due to their very low energy (entry 4). Based on these results, the use of Ø 5 mm balls was the best choice. By increasing the number of Ø 5 mm balls, **4** selectivity can be further improved (entries 5, 6). Although 40 pcs afforded close to exclusive formation of **4**, 35 pcs were used in further measurements to make the effect of the other reaction parameters visible. Thus, when the reaction mixture was milled for half of the previously used time, both the conversion and **S4** decreased (entry 7).

**Figure 1:**
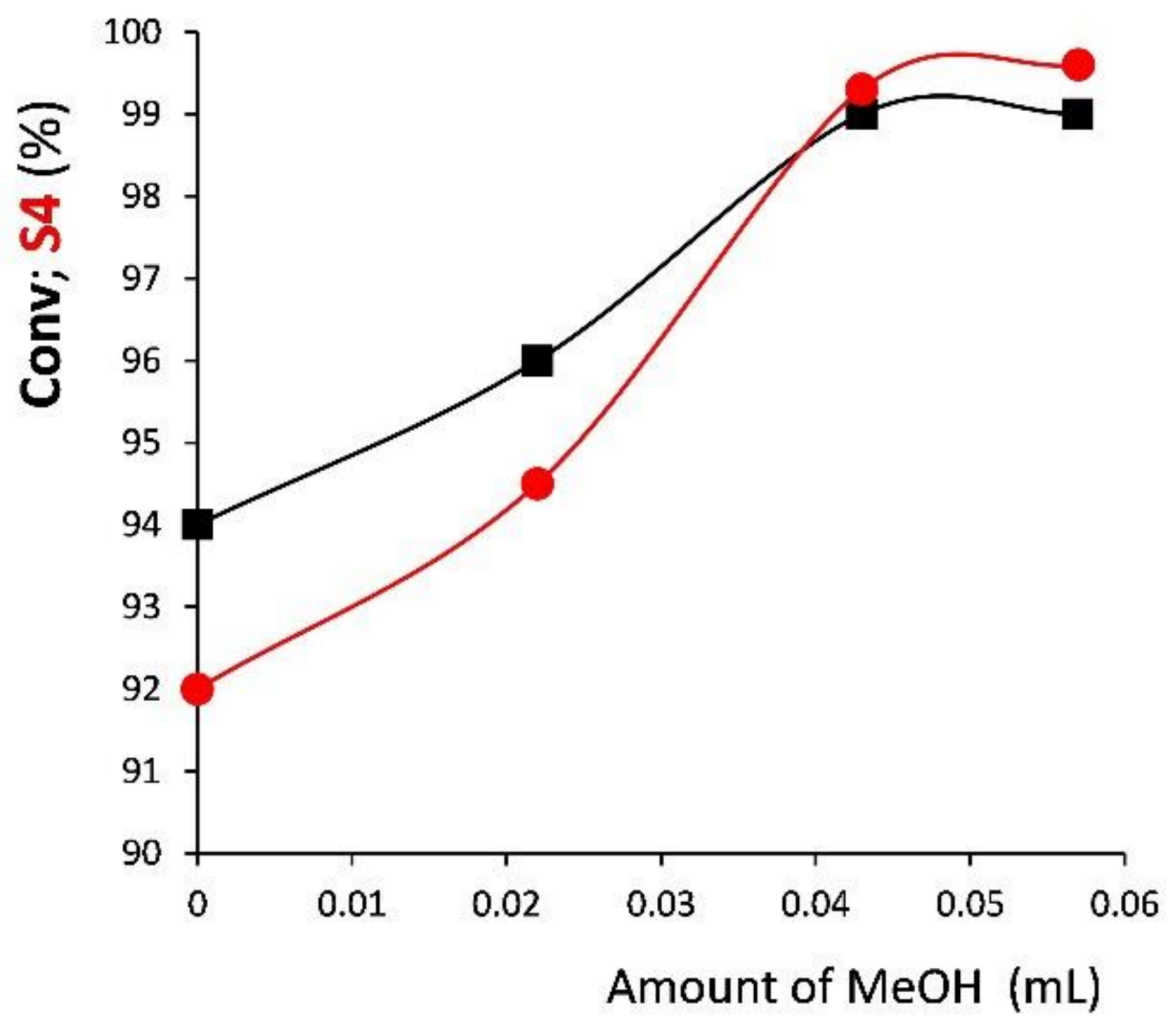
Effect of MeOH amount (■: **Conv**, conversion; ●: **S4**, selectivity of **4**—both determined by GC-FID). Reaction condition: 100 mg Amberlyst^®^ 15, 1 mmol **1**, 1.5–1.1 mmol **2**, total liquid volume of 0.213 mL, 35 pcs Ø 5 mm ZrO_2_ balls, 30 Hz, 180 min.

Our results obtained in batch reactions showed that a small amount of MeOH may improve the selectivity of **4** in this cascade reaction. Moreover, many of the mechanochemical reactions that have been reported up to now are not completely neat; a small amount of additional liquid with an energy mediating role is often used, which also ensures the proper mixing of the system [79,80]. By decreasing the excess of **2** and replacing the missing volume with MeOH, conversion and **S4** of the mechanochemical reaction were improved (Figure 1). With as little as 1.1 eq **2** and 0.057 mL MeOH, the reaction solely afforded product **4** in the mixer mill following 180 min of grinding.

Decreasing either the milling time or frequency had an unfavourable effect in reactions using 100 mg Amberlyst^®^ 15, 0.057 mL MeOH and 35 pcs of Ø 5 mm grinding balls. Thus, at least 180 min of milling at 30 Hz was necessary to achieve high conversion and up to 99% **S4** (Table 4). Importantly, the reaction time can be decreased significantly compared to the magnetically stirred system (3 vs. 24 h). Although the reaction is shorter, more catalyst is demanded to achieve a good result; 50 mg Amberlyst^®^ 15 was not enough to promote the second ring-closing step properly (Table 4, entry 7). The use of pre-milled Amberlyst^®^ 15P (entry 3, 5) gave similar results to the commercial form in mechanochemical reactions, probably due to grinding of the bead-like Amberlyst^®^ 15 during the initial stages of the milling process, with pre-milling thus losing its importance.

Finally, a few other acidic Brønsted catalysts used in the batch system were also re-examined under mechanochemical conditions (Table 5). The reaction carried out without a catalyst gave low conversion and **S4** (entry 1). Although the system warms up during the milling process, i.e., the mixture is heated up to *cca* 55 °C after 3 h of milling (based on our previous studies) [100], the temperature in the jar is not high enough to promote either step. The best results so far were provided by the Amberlyst^®^ 15 used in this study (entry 2). In the magnetically stirred system, Amberlyst^®^ XN-1010 also gave similar results; however, the mechanochemical reaction was less efficient than with the former, probably due to its lower ion-exchange capacity (Amberlyst^®^ 15: 4.7 meq/g, Amberlyst^®^ XN-1010: 3.3 meq/g [101,102]; entry 3). Thus, a higher amount of this material should be used. Although Nafion™ NR50 gave a similar result to the Amberlyst^®^ resins in batch reactions at 90 °C, in the ball-milled reaction the former was much less efficient. Additionally, with the lower temperature reached in the mechanochemical system, the non-porous (and having low surface area) Nafion™ NR50 polymer was not brittle and consequently was not ground into powder. Instead, this material formed a thick, sticky paste which was not mixed properly with the reactants. The acid-treated montmorillonite (Mont K-10) mostly catalyzed the first ring-closing step in the mixer mill, which may also be attributed to the low temperature of the mixture in the milling jars.

## 3. Materials and Methods

The anthranilamide (**1**), ethyl levulinate (**2**), *p*-toluenesulfonic acid monohydrate (*p*-TsOH) and the applied methanol were obtained from commercial sources (Sigma-Aldrich, St. Louis, MO, USA) and used as received. The heterogeneous acid catalysts were commercial materials: Amberlyst^®^ 15 (Sigma-Aldrich, St. Louis, MO, USA; brown-grey beads, ion-exchange capacity: 4.7 meq/g, average pore diameters 265 Å, surface area: 45–55 m^2^/g, [101,102,103]); Amberlyst XN-1010 (Sigma-Aldrich Chem, Steighem, Germany, presently not available; dark grey beads, ion-exchange capacity: 3.3 meq/g, average pore diameters 51 Å, surface area: 540 m^2^/g [101,102]); Nafion™ NR50 (Sigma-Aldrich, St. Louis, MO, USA, has been discontinued; opaque white pellet, ion-exchange capacity: ≥0.8 meq/g, non-porous, surface area: <0.02 m^2^/g [101]); Deloxan^®^ ASP (Degussa AG, Hanau, Germany; white powder, 0.80 mmol S/g [104]); montmorilonite K10 (Mont K10, Sigma-Aldrich, St. Louis, MO, USA) (light beige powder, surface area: 220–270 m^2^/g, total concentration of the acid centers 0.45 mmol/g [105]); montmorilonite KSF (Mont KSF, Sigma-Aldrich, St. Louis, MO, USA, currently is not available; off-white powder, surface area: 20–40 m^2^/g [106]); and Silica gel 60 (Merck Millipore, Darmstadt, Germany, white powder, particle size: 250–500 μm, pore size: 150 Ǻ, pore volume: 1.15 mL/g). In some reactions, Amberlyst^®^ 15 was used after pre-milling for 10 min with 30 Hz agitation frequency in a 10-mL ZrO_2_ grinding jar applying 35 pcs of Ø 5 mm ZrO_2_ grinding balls (Retsch GmbH, Haan, Germany).

^1^H and ^13^C NMR spectra of the purified products were recorded on a Bruker Ascend 500 instrument using CDCl_3_ solvent. Products were isolated by crystallization in ethyl acetate (**4**) or purified by flash chromatography (**3**). Gas-chromatographic analysis of the reaction products was carried out using an Agilent Techn. 6890 N GC-5973 MSD (GC-MSD, Agilent Co., Santa Clara, CA, US) equipped with a 30 m long HP-1MS capillary column for mass spectrometric identification of the products. For quantitative analysis, an Agilent 7890A GC-FID (GC-FID, Agilent Co., Santa Clara, CA, US) chromatograph equipped with a capillary column (HP-5 30 m, J & W from Agilent Co., Santa Clara, CA, US) was used.

### 3.1. Reaction of Anthranilamide and Ethyl Levulinate in the Batch System: General Procedure

The reactions were carried out in 4-mL closed glass vials immersed in a heated oil bath and the slurries were stirred magnetically (600 rpm). In a typical reaction, 1 mmol **1**, 1.1–1.5 mmol **2** (0.022–0.057 mL MeOH) and the chosen catalyst were introduced into the vial and stirred at 60 or 90 °C for 24 or 48 h. Following the reactions, the products were dissolved in 3 mL MeOH, and the catalysts were separated by filtration or centrifugation. The liquid phases were analyzed by gas-chromatography using *n*-decane as the internal standard (GC-MSD and GC-FID). Conversions (**Conv**) and selectivities (**S3** and **S4**) were calculated based on the relative concentrations determined from chromatograms using the formulae given in the Supplementary Materials. The products that resulted in a few reactions were purified either by crystallization in ethyl acetate (**4**) or by flash chromatography using hexane/ethyl acetate 1/1 as eluent (**3**) for the determination of the yields. The identity of the isolated products was confirmed by ^1^H and ^13^C NMR spectroscopic measurements using CDCl_3_ as a solvent. The experiments were repeated at least 3 times and the reproducibility of product composition was found to be within ±1%.

### 3.2. Reaction of Anthranilamide and Ethyl Levulinate by Ball Milling: General Procedure

The reactions were carried out in 10-mL closed grinding jars with a ZrO_2_ inner coat and ZrO_2_ grinding balls (Ø 3, 5, 12, 15 mm). In a typical reaction, 1 mmol **1**, 1.1–1.5 mmol **2** (0.022–0.057 mL MeOH) and the chosen catalyst were introduced into the jars, and the chosen number of balls was then added to the system. The closed jars were placed into a Retsch MM400 mixer mill (Retsch GmbH, Haan, Germany) and agitated at a 30 Hz frequency for a maximum of 180 min. Following reactions, the products were dissolved in 1 mL MeOH, the jars and balls were washed twice with 1 mL MeOH and the unified liquid phase was filtrated and analyzed in the same manner as the products of the magnetically stirred reactions. The experiments were repeated at least 3 times and the reproducibility of product composition was found to be within ±1%.

Analytical data of the products:

Ethyl 3-(2-methyl-4-oxo-1,2,3,4-tetrahydroquinazolin-2-yl)propanoate (**3**) 

Flash chromatographic separation, eluent: hexane/ethyl acetate 1/1, off-white crystals, mp 88–91 °C. 

GC-FID analysis (HP-5 column): R_t_= 47.6 min.
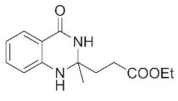


GC-MSD *m/z* (rel. int.): *262*(M^+^, 1), *201*(24), *173*(8), *161*(100), *119*(24), *92*(16), *65*(5), *42*(4).

^1^H NMR (500 MHz, CDCl_3_) δ (ppm): 7.85 (d, 1H, *J* 7.7 Hz, Ar-H), 7.27 (m, 1H, Ar-H), 6.90 (s, 1H, N-H), 6.78 (t, 1H, *J* 7.7 Hz, Ar-H), 6.58 (d, 1H, *J* 8.0 Hz, Ar-H), 4.34 (s, 1H, N-H), 4.10 (q, 2H, *J* 7.1 Hz, CH_2_), 2.65 (m, 2H, CH_2_), 2.10 (m, 2H, CH_2_), 1.54 (s, 3H, CH_3_), 1.20 (t, 3H, *J* 7.1, CH_3_).

^13^C NMR (125 MHz, CDCl_3_) δ (ppm): 173.7, 164.4, 145.9, 134.0, 128.3, 118.6, 114.5, 114.1, 69.9, 60.8, 36.6, 29.1, 29.0, 14.1.

3a-Methyl-2,3,3a,4-tetrahydropyrrolo[1,2-*a*]quinazoline-1,5-dione (**4**)

Separation by crystallization in ethyl acetate, white crystals, mp 162–165 °C.

GC-FID analysis (HP-5 column): R_t_ = 52.4 min.
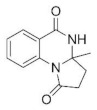


GC-MSD *m/z* (rel. int.): *216*(M^+^, 6), *201*(100), *1173*(35), *161*(6), *132*(6), *90*(6), *42*(2).

^1^H NMR (500 MHz, CDCl_3_) δ (ppm): 8.16 (d, 1H, *J* 8.1 Hz, Ar-H), 8.07 (d, 1H, *J* 7.8 Hz, Ar-H), 7.89 (s, 1H, N-H), 7.59 (m, 1H, Ar-H), 7.29 (m, 1H, Ar-H), 2.70 (m, 2H, CH_2_), 2.39 (m, 2H, CH_2_), 1.57 (s, 3H, CH_3_).

^13^C NMR (125 MHz, CDCl_3_) δ (ppm): 171.6, 163.3, 135.8, 133.8, 128.3, 125.0, 120.7, 119.5, 76.7, 74.5, 32.9, 30.0, 26.9.

## 4. Conclusions

In the present study, we have developed a method to increase the sustainability of the preparation of a pyrrolo[1,2-*a*]quinazoline-1,5-dione derivative through a one-pot cascade reaction occurring by reacting anthranilamide and ethyl levulinate. The application of a heterogeneous acid catalyst and mechanochemical activation in the two-step reaction was successful, resulting in a decrease in the necessary reaction time from 24 h in a magnetically stirred batch system to as little as three hours. The reaction was carried out in a ball mill via liquid-assisted grinding with only a minor excess of ethyl levulinate and using a slight amount of methanol. A polystyrene-based sulfonic acid-functionalized catalyst, i.e., Amberlyst^®^ 15, provided the best results both in the batch and in the mechanochemical reactions. In the latter system, the use of an acid catalyst with appropriate properties was essential, as the heat generated by the collision and friction of the grinding media was not sufficient to promote the second ring-closing step thermally.

## Figures and Tables

**Table 1:** Effect of the catalyst in the reaction of **1** and **2** ^a^.

Entry	Catalyst	Catalyst Amount	T (°C) ^b^	t (h) ^c^	Conv (%) ^d^	S3 (%) ^e^	S4 (%) ^e^
1	‒	‒	60	24	40	83	15
2	‒	‒	90	24	95	73 (60) ^f^	24
3	Silica gel 60	125 mg	90	48	99	48	51
4	Mont K10	125 mg	90	24	>99	60	39
5	Mont K10	125 mg	90	48	>99	35	63
6	Mont KSF	125 mg	90	48	>99	40	58
7	*p*-TsOH	5 mol%	60	24	>99	48	51
8	*p*-TsOH	5 mol%	90	24	>99	‒	99 (90) ^f^
9	Deloxan^®^ ASP	100 mg	60	24	>99	80 (70) ^f^	19
10	Deloxan^®^ ASP	125 mg	90	24	>99	‒	98
11	Nafion™ NR50	86 mg (2 pcs)	60	24	96	77 (61) ^f^	21
12	Nafion™ NR50	84 mg (2 pcs)	90	24	>99	‒	99 (88) ^f^
13	Amberlyst^®^ XN-1010	100 mg	60	24	>99	‒	99 (90) ^f^
14	Amberlyst^®^ 15	100 mg	60	24	>99	‒	99 (91) ^f^

^a^ Reactions were carried out using 1 mmol **1** and 1.5 mmol **2** with magnetic stirring (600 rpm). ^b^ Reaction temperature. ^c^ Reaction time. ^d^ Conversion of **1** determined by gas chromatography (GC-FID). ^e^ Selectivities of products **3** and **4** were determined by GC-FID, the selectivities of other products were 1–3%. ^f^ In parentheses, the yield of the corresponding product following purification by flash chromatography (**3**) or by crystallization in ethyl acetate (**4**).

**Table 2:** Effect of **2** and methanol amount on the results obtained in the reaction of **1** and **2** ^a^.

Entry	EL Amount (eq)	MeOH Amount (μL)	Conv (%) ^b^	S3 (%) ^c^	S4 (%) ^c^
1	1.5	‒	>99	35	64
2	1.1	‒	>99	55	42
3	1.1	57	>99	1	97 (89) ^f^
4 ^d,e^	1.1	57	‒	96	2
5 ^e^	1.1	57	‒	‒	99 (91) ^f^

^a^ Reactions were carried out using 50 mg pre-milled Amberlyst^®^ 15P and 1 mmol **1** at 60 °C for 24 h. ^b^ Conversion by GC-FID. ^c^ Selectivities of **3** and **4** by GC-FID. ^d^ Reaction without catalyst. ^e^ Using 1 mmol **3** as the starting material (isolated by flash chromatographic purification of previously obtained reaction mixtures). ^f^ In parentheses, the yield of crystallized **4**.

**Table 3:** Effect of the size and number of grinding balls in the reaction of **1** and **2** ^a^.

Entry	Diameters of Balls (mm)	Number of Balls (pcs)	Conv (%) ^b^	S3 (%) ^c^	S4 (%) ^c^
1	15	1	98	36	62
2	12	1	98	54	42
3	5	25	97	13	85
4	3	125	>99	90 (80) ^d^	8
5	5	35	98	4	94
6	5	40	>99	‒	99 (92) ^d^
7 ^e^	5	35	77	78	20

^a^ Reactions were carried out using 100 mg Amberlyst^®^ 15, 1 mmol **1** and 1.5 mmol **2** with an agitation speed of 30 Hz for 180 min. ^b^ Conversion by GC-FID. ^c^ Selectivities of **3** and **4** by GC-FID. ^d^ In parentheses, the yield of the purified **3** or **4**. ^e^ 90 min reaction.

**Table 4:** Effect of milling time and agitation frequency in the reaction of **1** and **2** ^a^.

Entry	Reaction Time (min)	Agitation Frequency (Hz)	Conv (%) ^b^	S3 (%) ^c^	S4 (%) ^c^
1	60	30	90	58	40
2	120	30	93	28	70
3 ^e^	120	30	97	25	73
4	180	30	98	‒	99 (88) ^d^
5 ^e^	180	30	>99	‒	99 (92) ^d^
6	180	20	99	80	18
7 ^f^	180	30	99	38	60

^a^ Reactions were carried out using 100 mg Amberlyst^®^ 15, 1 mmol **1**, 1.1 mmol **2** and 57 μL MeOH with 35 pcs of Ø 5 mm ZrO_2_ grinding balls. ^b^ Conversion by GC-FID. ^c^ Selectivities of **3** and **4** by GC-FID. ^d^ In parentheses, the yield of the crystallized **4**. ^e^ Reaction using 100 mg pre-milled Amberlyst^®^ 15P. ^f^ Using 50 mg Amberlyst^®^ 15.

**Table 5:** Influence of the catalyst in the mechanochemical reaction of **1** and **2** ^a^.

Entry	Catalyst	Catalyst Amount	Conv (%) ^b^	S3 (%) ^c^	S4 (%) ^c^
1	‒	‒	5	80	18
2	Amberlyst^®^ 15	100 mg	98	‒	99
3	Amberlyst^®^ XN-1010	100 mg	99	14	85
4	Nafion™ NR50	85 mg (2 pcs)	48	63	35
5	Mont K10	125 mg	52	82	16

^a^ Reactions carried out using 1 mmol **1**, 1.1 mmol **2** and 57 μL MeOH with 35 pcs Ø 5 mm ZrO_2_ grinding balls at 30 Hz for 180 min. ^b^ Conversion by GC-FID. ^c^ Selectivities of **3** and **4** by GC-FID.

## Data Availability

The data presented in this study are available on request from the corresponding author.

## Supplementary Materials

The following supporting information can be downloaded at: https://www.mdpi.com/article/10.3390/molecules27175671/s1, General formulae; Figure S1: Effect of temperature on the reaction of anthranilamide (**1**) with ethyl levulinate (**2**) in the batch system; Figure S2: Effect of the catalyst amount on the reaction of anthranilamide (**1**) with ethyl levulinate (**2**) in the batch system; ^1^H and ^13^C NMR spectra of the isolated products; Chromatograms and mass spectra of the products.

## Sample Availability

Not available.
