# Supplementary material for: Synthesis of a Pyrrolo[1,2-a]quinazoline-1,5-dione Derivative by Mechanochemical Double Cyclocondensation Cascade
Source: Molecules. 2022 Sep 2;27(17):5671. doi: 10.3390/molecules27175671 (PMC9478961; doi:10.3390/molecules27175671)

Supplementary Material

Synthesis of a Pyrrolo[1,2-*a*]quinazoline-1,5-dione Derivative by Mechanochemical Double Cyclocondensation Cascade

Vanessza Judit Kolcsár [1] and György Szőllősi [2,*]

[1] Department of Organic Chemistry, Institute of Chemistry, Faculty of Science and Informatics, University of Szeged, Dóm tér 8, 6720, Szeged, Hungary

[2] ELKH-SZTE, Stereochemistry Research Group, University of Szeged, Eötvös utca 6, 6720, Szeged, Hungary

* Corresponding author

Content

		page
General formulae		2
Figure S1.	Effect of temperature on the reaction of anthranilamide (**1**) with ethyl levulinate (**2**) in the batch system	3
Figure S2.	Effect of the catalyst amount on the reaction of anthranilamide (**1**) with ethyl levulinate (**2**) in the batch system	3
^{1}H and ^{13}C NMR spectra of the isolated products		4
Chromatograms and mass spectra of the products (GC-FID and GC-MSD)		6

General formulae

Reaction of anthranilamide (**1**) and ethyl levulinate (**2**):

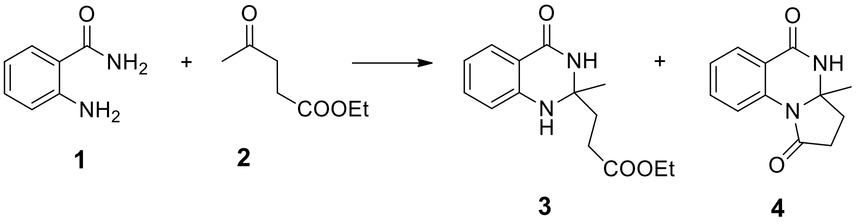

The conversions and the selectivities were calculated with the formulas given below using the relative concentrations determined by gas chromatography.

$$\mathbf{Conv} = \frac{c_{0,\mathbf{1}} - c_{\mathbf{1}}}{c_{0,\mathbf{1}}} \times 100\ (\%)$$

$$\mathbf{S3} = \frac{c_{\mathbf{3}}}{c_{0,\mathbf{1}} - c_{\mathbf{1}}} \times 100\ (\%)$$

$$\mathbf{S4} = \frac{c_{\mathbf{4}}}{c_{0,\mathbf{1}} - c_{\mathbf{1}}} \times 100\ (\%)$$

where:
Conv: conversion, **S3**: selectivity of product **3**, **S4**: selectivity of product **4**;
$c_{0,1}$: initial concentration of **1**; c_1: concentration of the unreacted **1**;
c_3: concentration of the resulted **3**; c_4: concentration of the resulted **4**.

Effect of temperature on the reaction of anthranilamide (1) with ethyl levulinate (2) in the batch system

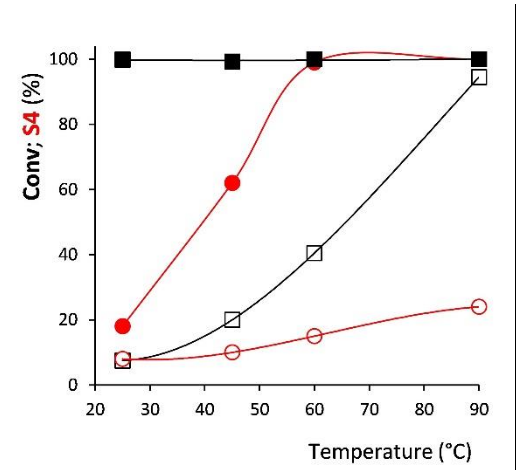

Figure S1. Effect of the reaction temperature with (■, ●) and without (□, ○) catalyst. Reaction conditions: 100 mg as received Amberlyst® 15, 1 mmol anthranilamide (**1**), 1.5 mmol ethyl levulinate (**2**), 24 h, magnetic stirring 600 rpm; conversion: ■, □; selectivity of **4** (S4): ●, ○.

Effect of the catalyst amount on the reaction of anthranilamide (1) with ethyl levulinate (2) in the batch system

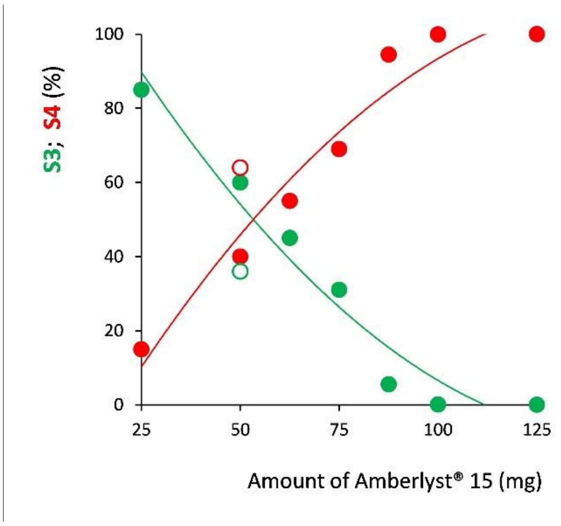

Figure S2. Effect of the catalyst amount on the selectivity of **3** (S3: ●) and selectivity of **4** (S4: ●) using as received Amberlyst® 15, and on the selectivity of **3** (S3: ○) and the selectivity of **4** (S4: ○) using pre-milled Amberlyst® 15; Reaction conditions: 1 mmol **1**, 1.5 mmol **2**, 60°C, 24 h, magnetic stirring 600 rpm, the conversion of **1** was >99% in all these reactions.

^{1}H and ^{13}C NMR spectra of the isolated products

Ethyl 3-(2-methyl-4-oxo-1,2,3,4-tetrahydroquinazolin-2-yl)propanoate (**3**)

^{1}H NMR (500 MHz, $CDCl_3$)

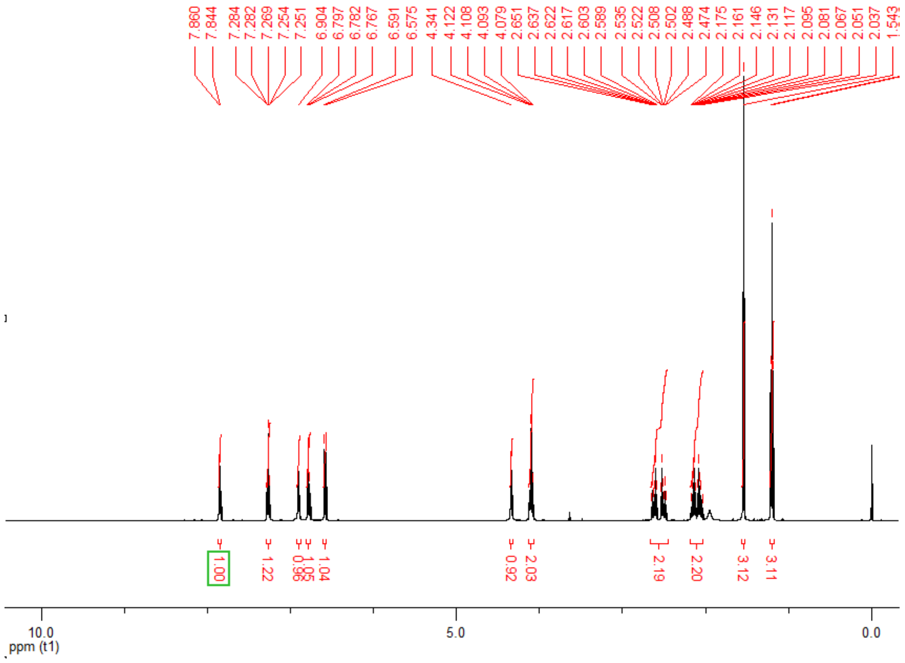

^{13}C NMR (500 MHz, $CDCl_3$)

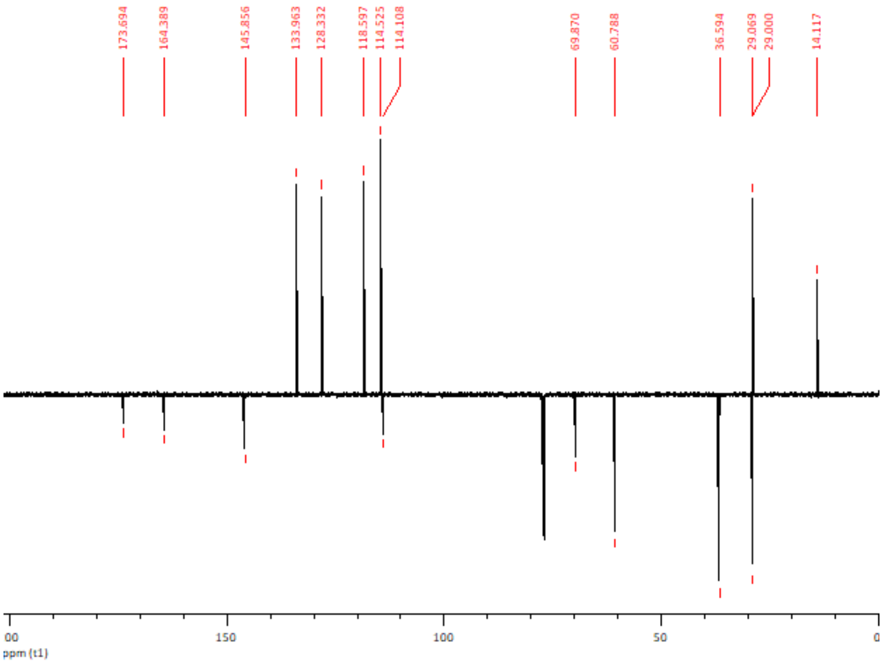

3a-Methyl-2,3,3a,4-tetrahydropyrrolo[1,2-*a*]quinazoline-1,5-dione (**4**)

^{1}H NMR (500 MHz, $CDCl_3$)

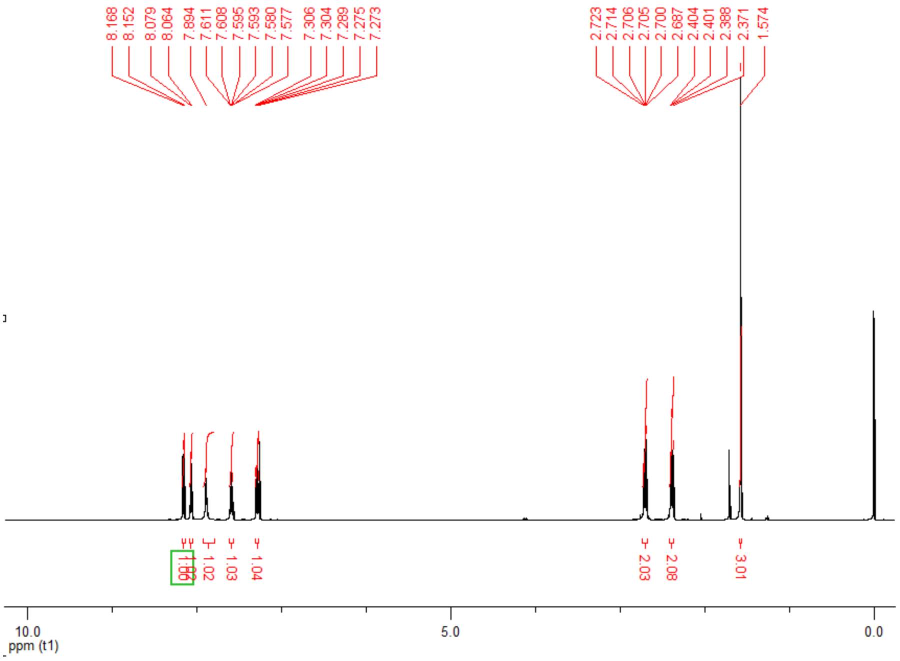

^{13}C NMR (500 MHz, $CDCl_3$)

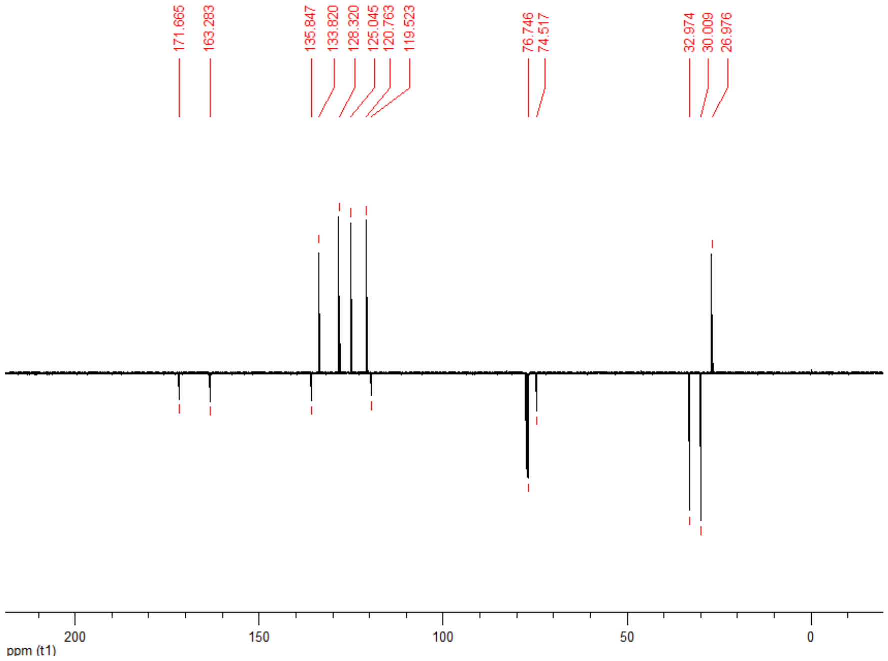

Chromatograms and mass spectra of the products (GC-FID and GC-MSD)

Separation of the products using GC-FID, chromatograms of raw products

Table 5, entry 4

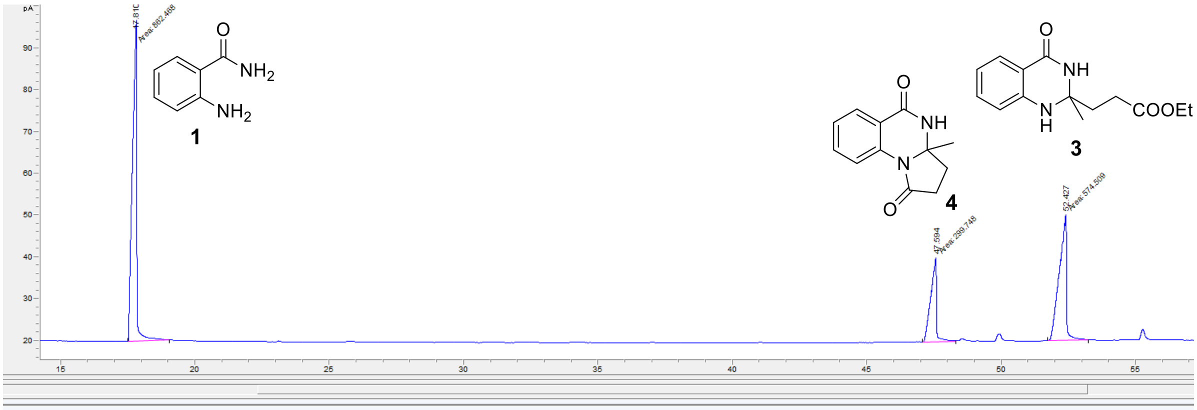

#	Time	Area	Height	Width	Area%	Symmetry
1	17.81	862.5	76.9	0.1868	49.661	5.081
2	47.594	299.7	20.1	0.2483	17.259	6.004
3	52.427	574.5	30.2	0.3168	33.080	5.983

Table 2, entry 4

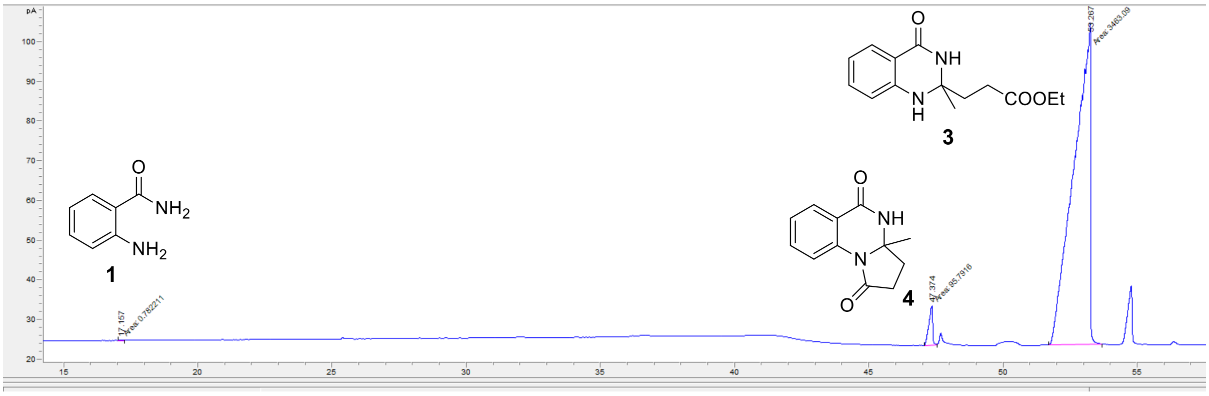

Table 2, entry 5

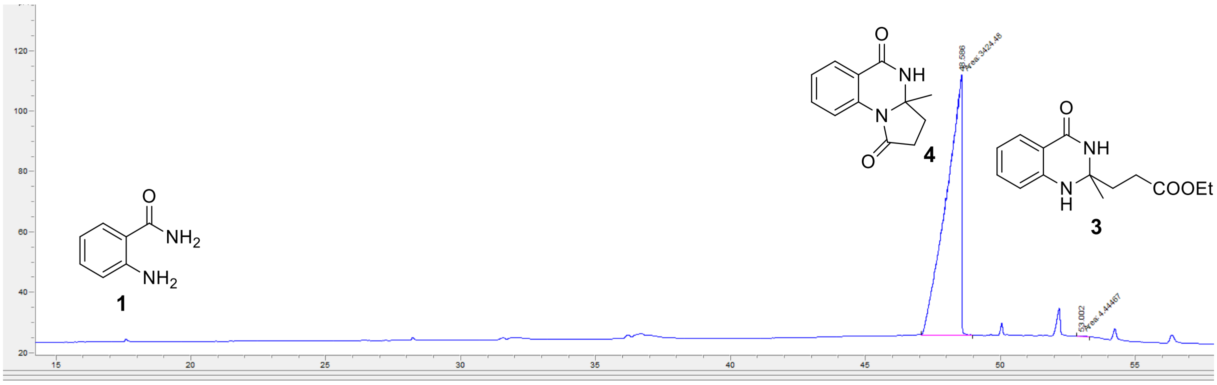

Retention times: R_t(**1**)= 17.8 min, R_t(**3**) = 47.6 min, R_t(**4**) = 52.4 min

Identification of the products by GC-MSD, EI-MS mass spectra of the purified products

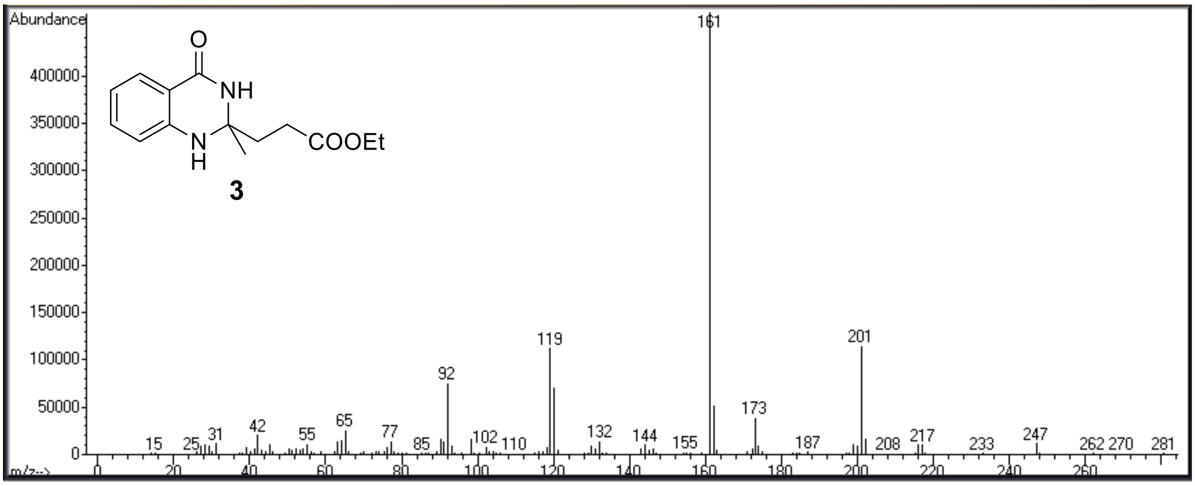

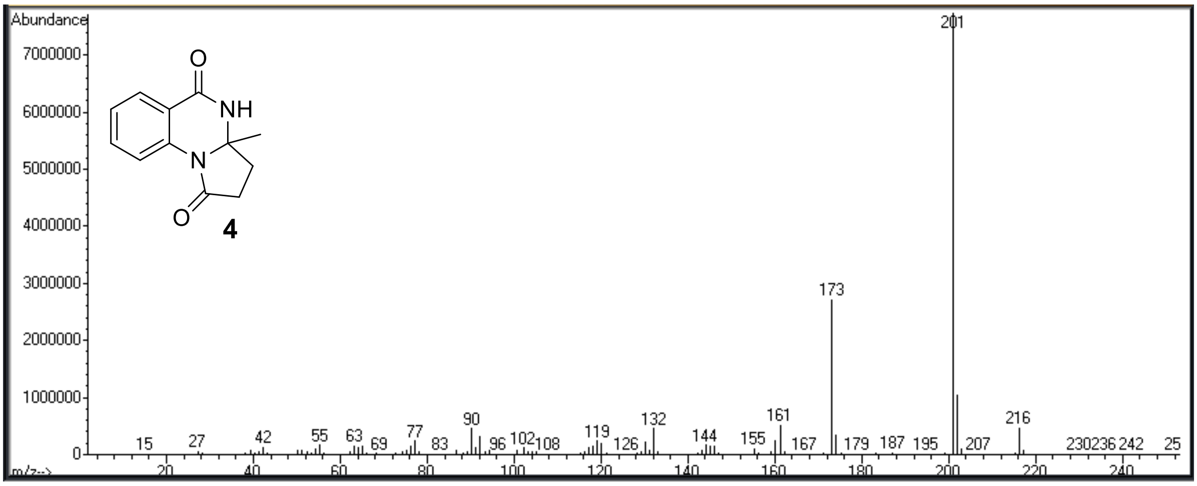

Supplement: Supplementary file 1 [file molecules-27-05671-s001.zip › molecules-1879553-SI.pdf]
